# Dr. Kentish, on the Treatment of Burns and Scalds

**Published:** 1806-07

**Authors:** G. Kentish

**Affiliations:** Bristol


### To the Editors of the Medical and Physical Journal.

Gentlemen,

In your 86th Number there are some Observations by Dr. Kinglake, on a paper I sent you respecting the treatment of burns; he seems principally to be offended with the certainty, or rather daringness of my prognosis. Had the Doctor read my first Essay on the subject, he would have seen the source from whence I drew my experience, an authority to which he owns " all must bow assent, when that experience has been unquestionable." I have there shewn that patients immersed in pure liquid fire, have by one treatment been exhausted by pain and fever in the space of nine days; that by another, existence was prolonged to the twelfth; and that by a third the patient was saved; and this, when all other circumstances, except the treatment, was alike. It is seldom a medical writer can so clearly prove the utility of his practice; circumstances happily occurred to me, to ascertain so important a point. I gave the result to my brethren, and am happy to think that in this age of deception, and in the delusive science of medicine, all practitioners who have tried it, and compared it with the other means generally recommended, have acknowledged its superiority, and the relations I have made to be founded on truth. Thus I have shewn the field I had for experience was extensive; and the conclusions fairly drawn from that experience, authorises me to make the assertion which has offended Dr. Kinglake.

Now let me refer to Dr. Kinglake's experience on this subject; he says, that an early and unremitted application of cold water has, in numerous instances of burns and scalds under his own direction, given all the aid that could either be wished for by the patient or practitioner, i. e. I suppose, that the patient so scalded or burnt, got well. Of this fact I shall not for a moment doubt; but what does it prove? merely that burns or scalds, of a certain extent, will get well in defiance of the application of cold water. It is not in these accidents alone I have known Nature triumphant over every officious interference of art, which have retarded her progress in re-establishing the equilibrium of health; no, but we must allow there are means by which she may be aided in her endeavours; for if I find in extreme cases, that death is the result of one mode, and life of another, I think I may fairly conclude, that in cases of less violence, the treatment treatment according to the *principle* laid down in extreme cases, must be the fittest; though I fully and freely believe, that cases have gotten well by very different means, though not so soon, or with so little pain. A gentleman was scalded, and he used Perkins's tractors; he got well, and recommended the tractors in all cases. Was he capable of judging? (would Dr. Kinglake recommend them?) Dr. Warwick, to convince him of their inutility, allowed both his hands to be scalded; the one was tractorised, and the other done nothing to; the pain of each was equal, and they went through the curative process in the same time. To please the patient or practitioner in the cure of a disease may be delusive, but to know that you have been instrumental in aiding Nature, requires a knowledge of her laws in that disease, and to know what she would have done without your assistance. When the comparative effects of different treatment in the same disease are fairly made, we may surely then expect to arrive at the first principles on which the treatment of such diseases ought to be conducted. In accidents of this kind I have traced their comparative effects, I have stated them in different Essays, and the mass of evidence from other practitioners, in support of the principles I have adopted are so convincing in my own mind, that in this department we have little more to do than generally to diffuse the knowledge of the facts already accumulated. That my practice is approved and diffused, I shall give some observations from a late American work: Dr. Redman Cox, in a publication he conducts, called "The Philadelphia Medical Museum," has given some account of my opinions on this subject, illustrated by some cases which strongly prove its efficacy, more particularly one in the second volume, which I am induced to quote.

"Ann M'Quicker, five years of age, living with Mr. Robert Smith, No. 28, South Second Street: On the 11th of January, 1805, her clothes, which were callico, took fire, whilst she was sitting by the stove, and were nearly burnt off her back; the child in great agony ran into the street, in the midst of the snow, and by the endeavours of some to put out the flames, the clothes were stripped off her, carrying with the cinders the cuticle and skin in some places to an extent of surface, whose circumference, five feet in length of string would barely surround. She was taken into a neighbour's house almost in a state of nakedness, and a most miserable spectacle, arising from the mixture of blood and half burnt cinder, &c. adhering to this extensively injured surface. A medical gentleman gnetleman then in the neighbourhood, recommended the application of cold milk to the burn, and laudanum to be used internally. As no advantage was derived from this, and as the child suffered the most violent pain, I was requested to see her, as I happened to be at one of the neighbours. Uncertain of meeting with the gentleman who had previously prescribed for the child, and anxious if possible to afford her some relief, I concluded to go, and found her worse than had been represented. She was suffering under the most violent chilly fits I ever witnessed, and seemed as if it would carry her off, and the pain of the burn appeared to be absorbed in the superior suffering of the chill, for she exclaimed continually, whilst her teeth were violently chattering in her head, "Cover me, cover me!"

"The burn extended from the right axilla to below the great trochanter of the same side, over the clavicle and breast, and beyond the sternum, in some parts over the abdomen beyond the linea-alba, and the upper parts of the pudenda; backwards, it extended to the spine, nearly in a right line down, and the greater portion of the inferior part of the arm was either raw or in blisters. The fire had extended its influence considerably beyond the boundary of the sore, as was evident from the erysipelatous blush, and from several small blisters scattered here and there. It appeared scarcely possible that so young a child should survive so extensive a wound, and even if the first violence of the injury was overcome, it remained doubtful if the copious suppuration would not be greater than she could support. Nevertheless, I felt persuaded that nothing could serve her but Kentish's plan, and I ordered immediately spirit of turpentine on rags to the whole surface of the sore; from the hurry I neglected to have it warmed, which would probably have aided in sooner removing the chill. In the mean time she took thirty drops of laudanum, and the rags were kept constantly wet till the liniment was prepared, as a permanent application. The laudanum was repeated, as the chill did not entirely subside, and the pain continued violent for several hours. She was ordered the use of wine and water to drink, and the liniment was applied anew at bed-time, and some more laudanum, which relieved her, but did not prevent a restless night. The following day, the 12th, the pain though great was much alleviated, and the liniment was continued with occasional doses of laudanum. She complained of great difficulty in swallowing, which was ascribed to the flame having been drawn into the fauces. 13th, Symptoms the same; same; slight appearance of suppuration; the blackness of the sore is now chiefly removed by the tinder, &c. adhering to the ointment, and coming away with the dressing. On the 14th, suppuration commenced, and became daily more copious; she was now ordered laxative medicines and put on a strict diet. One part was conspicuous of about five or six inches diameter, in the lower part of the abdomen, bounded by the pubes and linea alba, the ilium, and a line from thence to the navel, which seems to be so much destroyed that a deep slough may probably come away. As suppuration was now well advanced, I applied about the 16th, Turner's cerate, chiefly as an estimate of the value of either application in such an extensive burn. The result was, the child was worse all the day and night, with additional fever and pain, and considerable alteration in the healthy aspect of the sore. The liniment consequently was reapplied with great relief; her bowels were moved with senna and manna, and by the next day things were as favourable as before.

" 20th. Pain still very considerable from the great extent of the sore, which precludes the smallest motion without rubbing some part of it. She is obliged to be shifted bodily out of bed, in order to be dressed, which causes much pain, as she is forced to stand the whole time. The suppuration became now very copious, I therefore again tried Turner's cerate, and chalk finely levigated, but the alteration for the worse, in twenty-four hours, was so great, that I was in no hurry to renew the trial; the turpentine liniment again restored its healthy state. During all this time she was strictly dieted, and so highly excitable was her system, that from foolishly giving her a small piece of chicken for dinner, which she thought she could eat, a violent degree of fever took place, which kept her restless and uneasy all night, with augmented pain of sore; and when dressed in the morning, in place of fine healthy granulations and suppuration, an ugly ill-conditioned ulcer, with sanious discharge, presented itself to view. Purging and close attention to diet once more, in a short time, overcame this, and the edges were rapidly cicatrizing. It was now thought adviseable to get her into the hospital, as from the extent of the sore, it would take a great time to heal, and she was admitted January 31, 1805; in a short time it became necessary to order bark in decoction with jellies, &c. Serpentaria also, with other remedies calculated for the debilitated state of her stomach, were employed, by which means she recruited; the sore healed rapidly pidly under the use of Turner's cerate, so that by the middle of March, it was not larger than the palms of both hands, and confined to that part which I have stated to have been the most injured by the fire. A solution of white vitriol at this time hastened its cicatrization, and on the 11th of May she was discharged cured. Had she not accustomed herself to pick the sore, it probably would have been well two or three weeks sooner, as for four or five weeks previous to this it had not been more than from two to three inches in diameter.

"On measuring the circumference of the cicatrix before she left the hospital, it required a string of four feet six inches to surround it. I have above observed, that five feet would not have surrounded the extent of the injury sustained in the first instance, especially if that of the arm is included, which in the present measurement is not adverted to.

"The above instance, if I had had any doubts before of the effects of Kentish's plan of treating burns, would have completely silenced them; it gives me pleasure to add, that the physician who first prescribed for the patient, and who had had frequent opportunities of seeing very extensive injuries from burns and scalds, in the large plantations of some of the West India islands, was beyond measure gratified, at the powerful and rapid effects of the plan pursued above, which he said, he should have been fearful of using had he not been convinced of its mildness and utility by so striking an example."

This case is given by the Editor (Dr. Coxe) who adds in a note:

"I cannot, from a review of the above, and other cases of burns, which have come under my notice, but protest against the plan of Sir James Earle, of applying ice to burns, even of the most extensive nature. I have never seen his work on the subject, and can judge of it only by partial extracts."

The above case is too important, and too much to the point, to be allowed to pass without some observations. From the situation the child was in when Dr. Coxe first saw her, had he entertained the same ideas with Dr. Kinglake, I have little doubt but all vital action would have been extinguished in a short time, and it would then have been ranked with the many cases we hear of, where the patient is dreadfully burnt, and dies in a few hours. The too suddenly checking the violent action occasioned by the fire, by applying cold milk and cold air, had nearly extinguished life; the most violent efforts were made by the system to arouse itself from the fatal torpor, which by the the fortunate giving (by Dr. Coxe) both external and internal stimulants, was happily effected. The danger of suddenly checking an increased action may be observed in a variety of circumstances; the taking a draught of cold water when the body is heated, occasions sometimes the most dreadful effects; such as dimness of sight, staggering, difficult respiration, coldness of the extremities, imperceptible pulse, and, in the course of a few minutes, death.

But as I look upon the investigation of fist principles to be of the greatest consequence, and that we should cautiously avoid error; I feel obliged to Dr. Kinglake for opposing those I have laid down, as I trust his motives are pure; and as he has had considerable experience of the effects of cold water in such cases, I would be obliged to him if he would, in the next case that occurs to him, try my method, and candidly relate the result: I think he need not fear the safety of his patient, as not only my experience, but that of many of the most eminent practitioners in the kingdom, will warrant him in the experiment. The opinion of the physician who prescribed the cold milk, and who had seen in the West Indies many accidents of the kind, is of great value; he could form a comparison; his judgment is of itself a host; for had he not seen and been convinced of its mildness and utility, he, like many others, could not have conceived it possible; it is principally, because it shocks our early prejudices that my plan is thought harsh and terrible; but in reality it is the most mild and soothing, which under these circumstances could be imagined, and leads the easiest and readiest way to health. But to return to Dr. Coxe's case; the first part of the treatment until suppuration was successful beyond expectation; how he was disappointed in the use of cerat. lap. calamin. I am at a loss to account for, unless it was, as Mr. Harrold supposes may frequently be the case, that the ointment was *rancid*; when it ceases to be a mild application, and proves unpleasantly stimulating. This I should apprehend was the case, for it is afterwards mentioned, that the sore in the hospital healed rapidly under the use of the same cerate. The Doctor certainly did not give the chalk a fair trial; twenty-four hours was not enough to form a judgment from; as to not appearing so clean or healthy, it cannot be expected to have the same florid look, nor is it desirable it should; had the Doctor had more faith, and continued the chalk, I do not doubt he would have been as much pleased with its its effects, as he owns he is with the first part of the treatment. Should he have a further opportunity, being now in a state to form a comparison, I hope he will make it, and with the same candour he has used in the former relations. The utility of purging and a strict diet are beautifully illustrated by the latter treatment of the case, by which all the principles I have endeavoured to illustrate in my Essays are fully justified: and it is by experience like this I wish these principles to be tried. I am fully aware of the consequence of first principles; the effects of those I have endeavoured to elucidate, will not be confined merely to injuries from fire; they must influence the reasoner in a variety of morbid actions; and on that account merit a cautious and temperate investigation, by which their value may be duly appreciated. Had not my paper already appeared too long (owing to Dr. Coxe's case, which I did not think it fair to abridge), I should have sent you some other cases, strongly in favour of my plan; but these I refer to another opportunity.

I am, &c.

*Bristol, May* 10, 1806. G. KENTISH.

*P. S.* I beg Mr. Harrold to accept my thanks for the attention with which he has read my Essays; it is very satisfactory to an author, whose only wish is to convey information, (though by the bye, I fear it is not the wish of all medical writers) to know that he is understood. I congratulate him on the opportunity he has already had, and I hope he may have many more, to confirm and extend the principles which are so satisfactorily demonstrated.

